# A Clinically Relevant Functional Model of Type-2 Cardio-Renal Syndrome with Paraventricular Changes consequent to Chronic Ischaemic Heart Failure

**DOI:** 10.1038/s41598-020-58071-x

**Published:** 2020-01-27

**Authors:** Joanne Clare Harrison, Scott Duncan George Smart, Emma Maria Hinemoa Besley, Jessica Renee Kelly, Morgayn Iona Read, Yimin Yao, Ivan Andrew Sammut

**Affiliations:** Department of Pharmacology and Toxicology, School of BioMedical Sciences, University of Otago Medical School, Dunedin, New Zealand

**Keywords:** Cardiology, Medical research

## Abstract

Cardiorenal syndrome, *de novo* renal pathology arising secondary to cardiac insufficiency, is clinically recognised but poorly characterised. This study establishes and characterises a valid model representative of Type 2 cardiorenal syndrome. Extensive permanent left ventricular infarction, induced by ligation of the left anterior descending coronary artery in Lewis rats, was confirmed by plasma cardiac troponin I, histology and cardiac haemodynamics. Renal function and morphology was assessed 90-days post-ligation when heart failure had developed. The involvement of the paraventricular nucleus was investigated using markers of inflammation, apoptosis, reactive oxygen species and of angiotensin II involvement. An extensive left ventricular infarct was confirmed following coronary artery ligation, resulting in increased left ventricular weight and compromised left ventricular diastolic function and developed pressure. Glomerular filtration was significantly decreased, fractional excretion of sodium and caspase activities were increased and basement membrane thickening, indicating glomerulosclerosis, was evident. Interestingly, angiotensin II receptor I expression and reactive oxygen species levels in the hypothalamic paraventricular nucleus remained significantly increased at 90-days post-coronary artery ligation, suggesting that these hypothalamic changes may represent a novel, valuable pharmacological target. This model provides conclusive morphological, biochemical and functional evidence of renal injury consequent to heart failure, truly representative of Type-2 cardiorenal syndrome.

## Introduction

Cardiorenal syndrome (CRS) is a clinically recognised but poorly characterised condition which is generally divided into multiple subtypes to aid the selection of specialised interventions^1^. Type-2 CRS describes chronic cardiac dysfunction, such as chronic heart failure (CHF), leading to progressive chronic kidney disease (CKD). This worsening renal function can then accelerate the underlying myocardial dysfunction. Chronic kidney failure has been reported in 68.3% of heart failure (HF) patients, with reduced creatinine clearance (CrCl)^2^ and decreased estimated glomerular filtration rate (eGFR)^3^ being recognised predictive factors for mortality. Conversely, improved renal function promotes a favourable prognosis in HF^4^.

Current international clinical guidelines direct that CKD should be viewed as a prevalent condition of varying severity, and press the need for early detection and improved clinical management^5^. Pathological processes implicated in the generation of CKD secondary to HF include: haemodynamic parameters, excessive autonomic nervous system (ANS) efferent output, systemic release of endocrine mediators and proinflammatory cytokines, impairment of endogenous vasodilatory mechanisms, erythropoietin imbalances and anaemia^6–12^. Activation of systemic and renal renin–angiotensin aldosterone system (RAAS) results in increased angiotensin II (AngII) and aldosterone mediated effects on renal electrolyte and water retention, leading to plasma volume expansion and increasing myocardial workload^13^. Renal denervation has been shown to normalize renal angiotensin receptor expression in animal models of CHF and diabetic nephropathy^14,15^. Conversely, the dysfunctional kidney can release inflammatory cytokines into the systemic circulation, provoking myocardial damage. Renal injury is also indicated to increase sympathetic activity within the cardiovascular regulatory centres of the midbrain via the renal somatic afferent nerves, altering cardiac structure and function^16,17^. However, the existence of a clinical correlation between co-morbidities does not imply causality. To confirm causality, the direct effect of myocardial injury upon renal function needs to be replicated in a controlled environment, where possible cofounders can be minimized.

Many animal models of Type-2 CRS have utilized unilateral nephrectomy where the renal injury is surgically induced, rather than resulting as a consequence of the HF^18–20^. Other studies have reported changes in renal function following myocardial infarction (MI), however, there are no working models which produce both *de novo* renal functional and morphological changes, consistent with CKD directly consequent to chronic HF.

Current recommended treatments for HF, such as angiotensin converting enzyme (ACE) inhibitors and AT_1_R antagonists, mainly act to symptomatically treat the peripheral cardiovascular manifestations of HF. To date, the role of the cardiac control centres of the brain have largely been ignored in the search for new targets to treat HF. However, there is growing evidence that the central nervous system (CNS) plays a major role in the pathophysiology of ischaemic HF^21^. Severing the pathways connecting the organum vasculosum of the lamina terminalis to the paraventricular nucleus (PVN) of the hypothalamus was found to attenuate characteristic features of HF^22^ but the mechanisms of activation of the PVN in HF are yet to be clearly elucidated. Current theories include the systemic release of pro-inflammatory cytokines and Ang II from damaged myocardium, and an endothelial release of prostaglandin E_2_ acting directly on the PVN^23^. Cardiac neural afferent activation as a consequence of MI has also been directly implicated in the formation of pro-inflammatory cytokines in the PVN of the hypothalamus correlating with an increased activation of microglial cells in this region occurring independently of blood-borne pro-inflammatory cytokine levels^24–27^. Hypothalamic ROS production has also been associated with activation of the sympathetic system and may be implicated in the progression of renal and cardiac dysfunction in HF, as elevated levels of ROS have been observed in the PVN in HF^28–34^. We sought to examine the involvement of ROS as well as other neuroinflammatory mediators such as TNF-α in HF^33^.

Previous studies mostly examined PVN changes during the early stages of HF focusing on the four to six week period post coronary artery ligation with only one study examining this region at 56 days post. We therefore investigated if these biomarker changes in the PVN were maintained or even elevated over the course of HF pathogenesis. We also sought to determine if CHF simultaneously provoked changes in the hippocampal arm of the hypothalamic pituitary adrenal axis. Our current study characterizes a reliable animal model, truly representative of Type-2 CRS where chronic cardiac dysfunction at 90-days post-MI results in morphological and functional renal injury and indicates an ongoing involvement for the PVN in this syndrome. This model will enable a clearer definition of the pathology involved and the insights gained could be used to produce more targeted treatments.

## Results

### Confirmation of cardiac injury

**Figure 1:**
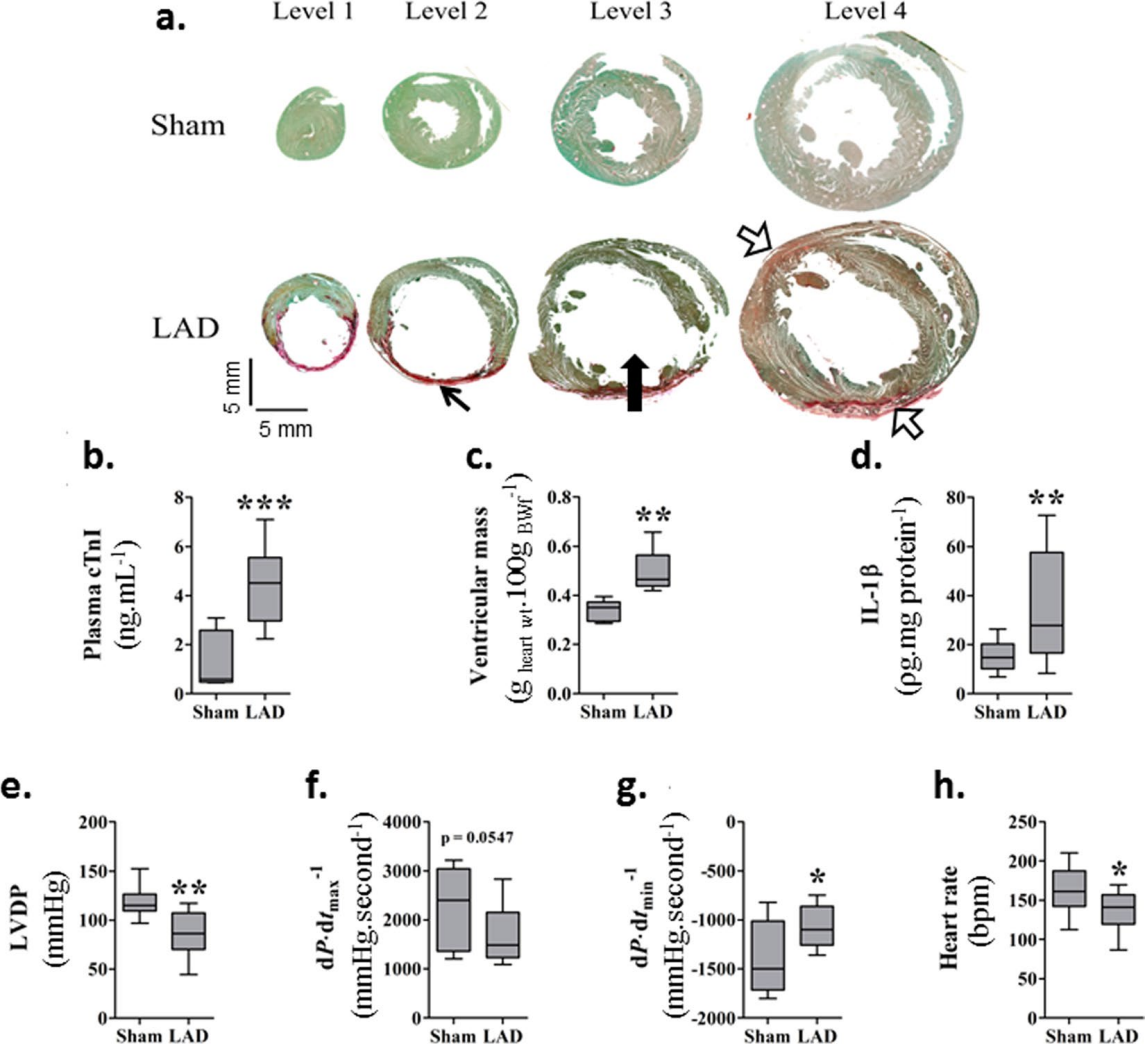
Confirmation of cardiac injury and functional loss 90-days post left anterior descending (LAD) coronary artery ligation: (**a**) Picrosirius-red stained cardiac sections showing ventricular enlargement (➔), wall thinning (↖) and a prominent red stained collagenous scar (⇨) in LAD artery ligated hearts compared to sham hearts. Ventricular apex is represented by level one with the base at level four. Box-Whiskers plots showing: (**b**) plasma troponin (cTnI) concentrations at 4 hr post-ligation, (**c**) left ventricular mass (g _heart wt_.100 g _BWf_^−1^), and (**d**) IL-1 β concentration in the myocardium 90-days post-ligation in sham and LAD animals respectively. Left ventricular function was measured as: (**e**) left ventricular developed pressure (LVDP), (**f**) systolic rate of contraction (d*P*.dt^−1^ max,), (**g**) diastolic rate of relaxation (d*P*.dt^−1^ min), and (**h**) un-paced heart rate (HR), in an isolated *ex vivo* preparation 90-days post-ligation in sham and LAD animals, respectively (*n* = 9 sham *n* = 9 LAD). *P < 0.05, ** *P* < 0.01, *** *P* < 0.001 compared to sham animals.

LAD ligation positioning was optimized in a preliminary study to produce a consistent high rate of LV myocardial scaring of the free wall (>20% wet weight) with acceptable perioperative mortality (<28%). There was no difference in whole animal fasted body weight (BW_f_) between surgical groups. Picrosirius-red staining revealed substantial LV enlargement at 90-days post-ligation, seen as an increased intraventricular volume compared to sham hearts, with substantial wall thinning and a prominent red stained collagenous scar (Fig. 1a). A significant elevation in plasma cTnI was seen at 4 hr post-ligation compared to sham animals (*P* < 0.05; Fig. 1b). LAD-ligated animals with cTnI levels <2 ng.ml^−1^ were excluded (3 animals) as the myocardial infarct was found to be ≤20% at termination (Supplementary Fig. 1 *Correlation between plasma cardiac troponin I (cTnI) at 4* *hr post coronary ligation and left ventricular infarct size (% free wall) measured at 90-days*), resulting in a total of 11 LAD animals surviving to 90-days included in the main study. Total ventricular mass per 100 g of BW_f_ was significantly (*P* = 0.0027) increased in LAD versus sham at the time of sacrifice (Fig. 1c). Average scar size in the LAD group was 24.2 ± 1.5% wet weight of the LV mass, including the septum. When individual regions of the myocardium were examined in a subset of rats (*n* = 4), it was found that LV and right free wall mass were significantly (*P* = 0.0103) increased following infarction (Table 1). IL-1β was significantly (*P* = 0.0082) increased in the peri-infarct region of LAD animals (Fig. 1d) at 90-days while no significant differences were seen in IL-4, IL-6 or IL-10 at this time point. Concentrations of IFN-γ, and TNF-α for all samples were below the detectable limits of the multiplex assay.

**Table 1:** Effect of LAD ligation on ventricular scar and wall size.

Tissue Region	Sham Mass (g)	LAD Mass (g)	*P*
Left ventricular free wall mass*	0.53 ± 0.10	1.03 ± 0.11	0.0103
Scar	0.0	0.32 ± 0.02	N.A.
Septum	0.42 ± 0.11	0.32 ± 0.05	0.2366
Right ventricular free wall	0.45 ± 0.04	0.58 ± 0.04	0.0381

Impact of left anterior descending coronary artery (LAD) ligation on ventricular regional mass at 90 days in a subset of animals (*n* = 4/group). *Including infarct scar.

Analysis of isolated LV haemodynamic function at 90-days showed that LAD ligation significantly (*P* = 0.0013) compromised LVDP (Fig. 1e) and rate of ventricular relaxation (*P* = 0.0141; Fig. 1g). Ventricular contractile rates (d*P*.dt min^-1^, Fig. 1f) in this surgically ligated group were also decreased (*P* = 0.0547). Spontaneous HR fell (*P* = 0.023) in the ligated animals (Fig. 1h) but no change in coronary flow between sham and LAD groups was observed.

### Blood analysis

Arterial blood samples taken at the time of LAD surgery and venous blood samples taken at organ harvest showed no evidence of anaemia, with no significant difference in hemoglobin concentration, or hematocrit, between sham and LAD animals. There was no significant change in pH, circulating electrolyte concentrations (Ca^2+^, Na^+^, or K^+^) or oxygen carrying potential of blood samples between groups or time points (see Supplementary Data Table 1).

### Impaired renal function and structural injury following CHF

**Figure 2:**
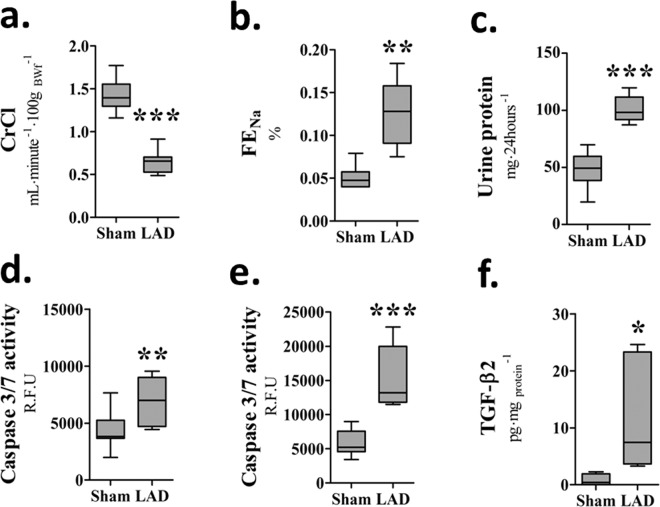
Left anterior descending (LAD) coronary artery ligation induces renal injury and functional deficits: (**a**) Calculated creatinine clearance (CrCl). ***P < 0.0001, *n* = 8/group. (**b**) Fractional excretion of sodium **(**FE_Na_). **P** = **0.0011, *n* *=* 6/group. (**c**) Urinary protein ***P < 0.0001, *n* = 9/group. (**d**) Caspase 3/7  activity in the renal cortex **P = 0.007, *n* = 8/group. (**e**). Caspase 3/7 activity in the medulla ***P = 0.0006, *n* = 7/group. (**f**) TGF-β2 concentration in the renal medulla *P = 0.0337, *n* = 5/group.

**Figure 3:**
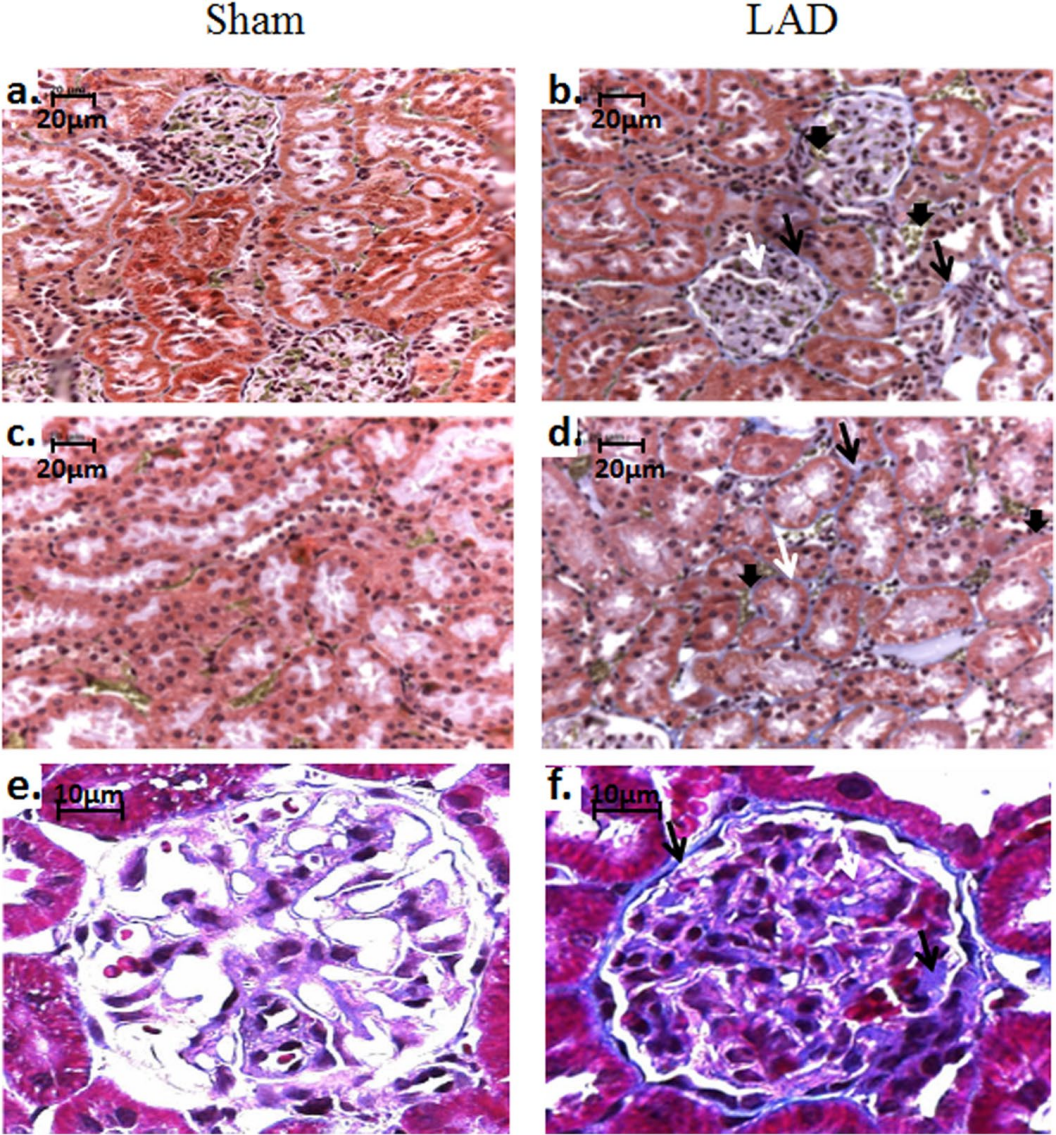
Left anterior descending (LAD) coronary artery ligation increases glomerular and tubulointerstitial collagen deposition and mesangial expansion. Martius Scarlet Blue stained renal cortex and medulla (**a–d**) Nuclei are stained blue/black, muscle pale red, collagen blue (➔), fibrin stained red, with early fibrin yellow, (↓) and old fibrin blue (⇨). (**a**) Renal cortex of a sham operated animal, showing normal morphology. (**b**) Renal cortex of a LAD-ligated animal, showing increased collagen deposition around glomeruli and in the tubulointerstitium. (**c**) Renal medulla of a sham operated animal, showing normal morphology. (**d**) Renal medulla of a LAD-ligated animal, showing increased tubulointerstitial collagen deposition. Masson’s trichrome stained renal glomeruli (**e, f**). Nuclei are stained blue/black, cytoplasm and muscle are stained red, and collagen is stained blue. (**e**) Glomeruli of a sham operated animal, showing normal morphology. (**f**) Glomeruli of LAD ligated animal, showing mesangial expansion (⇨) and increased collagen staining (➔).

At 90-days post-LAD all animals in the LAD-ligated group showed detrimental changes in renal function and structure. eGFR was decreased with CrCl reduced to 45% of the value of the Sham group (*P* < 0.0001; Fig. 2a), while FE_Na_ (*P* < 0.001; Fig. 2b) was significantly increased. Twenty four hour urinary protein levels were almost doubled (*P* < 0.0001) in the LAD-ligated animals (Fig. 2c). Caspase-3/7 activity was significantly increased in both the renal cortex (*P* = 0.007) and to a much greater extent in the medulla (*P* = 0.0006; Fig. 2d,e respectively). LAD surgery significantly (p < 0.05) increased IL-1β concentration in the kidney cortex, from 1.22 ± 0.14 to 2.11 ± 0.14 pg/mg protein in sham and LAD animals respectively. Elevated levels of profibrotic TGF-β2 were identified in the renal medulla (Fig. 2f), although the isoforms, TGF-β1 and TGF-β3, fell below the detectable limits of the assay at this 90-day time point. Increased collagen deposition was observed in the glomerular structures and in the tubulointerstitium of the LAD animals (Fig. 3). Glomerulosclerosis, seen as collagen deposition, was observed around the glomerular blood vessels and basement membrane. Additionally, interstitial fibrosis was also evident around the proximal, distal and convoluted tubule elements of the renal nephron. Basement membrane thickening and mild mesangial expansion were also seen in the LAD-ligated animals.

### Biochemical changes in the hypothalamic PVN and in the hippocampus following CHF

**Figure 4:**
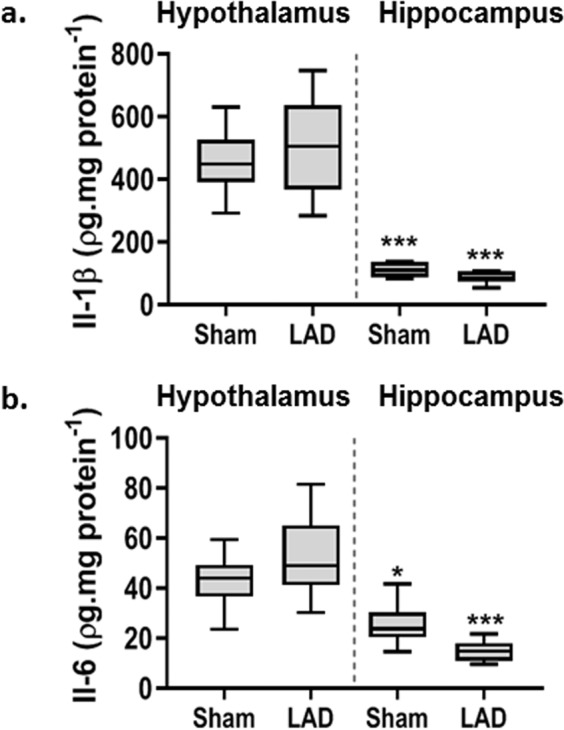
Cytokine levels in the Paraventricular Nucl us consequent to Chronic Ischaemic Heart Failure: (**a**) IL-1β and (**b**) IL-6 concentrations were elevated in the hypothalamus compared to control tissue from the hippocampus but no differences were observed between LAD-ligated animals *vs*. sham animals 90-days post-surgery. Data = mean ± SEM for each treatment group, *n* = 6–8/group. ***P < 0.001 compared to hypothalamus.

IL-1β and IL-6 levels were higher in the hypothalamic region assayed than in the reference hippocampal tissue (Fig. 4a,b). Neither cytokine was however, detectably altered within either brain region as a consequence of surgery. Levels of TNF-α were not detectable in these brain regions in either sham or following CHF development. Caspase-3/7 activity did not differ significantly across groups or between the two brain regions (data not shown).

**Figure 5:**
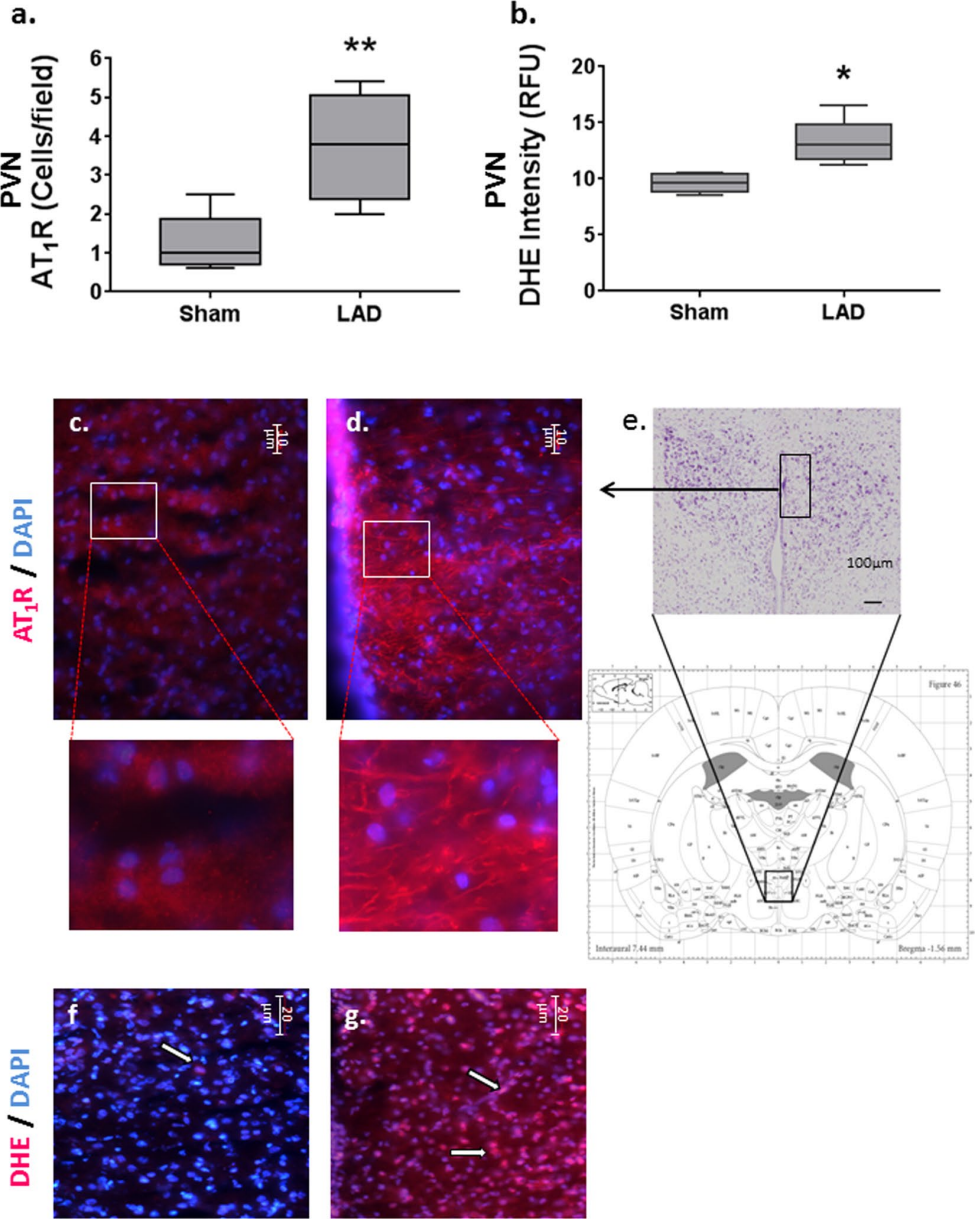
Paraventricular Nuclear changes consequent to Chronic Ischaemic Heart Failure: (**a**) AT_1_R expression in the paraventricular nucleus (PVN). The number of AT_1_R-positive cells per field counted in 6 sections per animal, *n* = 5 animals per treatment group. **P < 0.005 compared to sham group. (**b**) DHE fluorescence indicative of ROS production was measured in 4–6 sections per animal, *n* = 5/animals per treatment group. *P < 0.05 compared to sham group. Data is presented as mean ± SEM for each treatment group. AT_1_R localisation in the anterior PVN co-stained with the nuclear DAPI marker from (**c**) sham and (**d**) LAD ligated animals at 3 months post-surgery (scale bar = 10 µm) with inset figures representing a magnified (×3) subsection. Localisation of PVN in coronal sections (**e**) using anatomical landmarks, section stained with cresyl violet for illustrative purposes. Merged DHE fluorescent images showing ethidium co-localisation to DAPI-stained nuclei (indicated by ⇨) in the PVN of (**f**) sham showing DAPI nuclear staining and (**g**) LAD ligated animals with predominately DHE stained nuclear fluorescence at 3 months as a measure of ROS induced cleavage of DHE (scale bar = 20 µm).

AT_1_R expression associated with PVN neurons was increased (*P* < 0.006) in the LAD group (Fig. 5a,c,d). Localisation of the PVN in coronal sections was confirmed using anatomical landmarks (Fig. 5e). ROS production in the PVN was significantly elevated (*P* < 0.04; Fig. 5b) as indicated by the increased DHE nuclear staining in the LAD group (Fig. 5f,g). No significant differences were found in the number of Iba1 positive cells between LAD and sham groups (data not shown).

## Discussion

This study is the first to conclusively show that induction of a substantial myocardial infarct with impaired cardiac function, directly results in significant renal structural and functional impairment, by 90-days post-MI. This model, truly representative of Type-2 cardiorenal syndrome also implicates a role for ongoing elevated ROS in the PVN perpetuating the renal damage observed.

The increased LV mass following LAD artery ligation, with corresponding changes in rates of contraction and relaxation, pressure development and heart rate observed are all consistent with the development of CHF^35,36^. Cardiac dysfunction was accompanied by evidence of ventricular wall thinning and collagenous scar formation, helping confirm the development of diastolic heart failure. Gross morphological presentations associated with CHF were observed, including an increased ventricular mass compared to body weight due to enlargement of the LV free wall, combined with increased sphericity of the remodelled hearts^35,37,38^. A similar increase in heart to body weight ratio following MI has been seen in a Type-2 cardiorenal model^39^, however an earlier study had only reported an increase in LV mass in a small subset of animals^40^, possibly reflecting a difference in the severity of cardiac injury.

The model described here provides conclusive evidence that morphological, biochemical and functional glomerular and tubular injury results directly from a chronic primary cardiac insult truly representative of Type-2 CRS. Previous similar studies terminated at either 56 days^39^ or 112 days post-MI^41,42^ only produced a modest *de novo* change in renal morphology^39^ or increased fibrotic and injury markers^42^ secondary to MI but failed to demonstrate any significant changes in renal function. CrCl was shown to decrease mildly at 56 days post-LAD ligation^39^ and again in another 12 week CHF study^43^, however unlike the present study, no increase in FE_Na_, protein urea or tissue pathology were observed. Similarly, the 12 week study failed to demonstrate any changes in tubule function^43^. Our findings show that CrCl was significantly reduced at 90-day post-MI, indicative of impaired glomerular perfusion and filtration^44^ and suggests that the degree of glomerular injury recorded is directly related to the duration and extent of CHF development. The small increase in FE_Na_ our animals, reinforces the scenario of an early-stage renal decline existing at the termination point. Equally increased urinary protein indicative of tubular dysfunction observed in the LAD occlusion group 90-day post-MI is also consistent with the development of HF and is predictive of the progression of chronic renal disease to end-stage renal disease^45–48^. In contrast, the most realistic previously published model of Type-2 CRS lacked this predictive increase in microalbuminuria^43^. The detection of these functional differences in our findings may be attributable to the use of cTnI as a marker of cardiac injury, allowing us to limit our assessments to those animals with severe sizable infarcts. This refinement is in contrast to the limited histological measurements of infarct size used in the 12 week study. Our observation that the degree of renal injury is proportional to the extent of MI has been seen previously but required a combined insult of diabetes and MI to produce renal functional impairment^49^.

Renal apoptosis has been shown to progressively increase in animal models up to 56 days following MI and has also been clinically attributed to the pathological homeostatic signalling seen in CHF^39,50,51^. The elevated renal caspase 3/7 activity observed here at 90-days post-MI indicates that renal apoptosis is still ongoing at this later time point. The finding of proteinuria suggests the development of podocyte injury which is known to trigger fibrosis^52^. A profibrotic model of renal injury developing by 90-days post-MI is indicated here given the elevated levels of renal TGF-β2 detected^39,41^. Morphological changes, consistent with fibrotic remodelling subsequent to apoptosis, were confirmed through histological analysis showing increased collagen deposition in the tubulointerstitium and in the glomerular space, consistent with glomerulosclerosis.

Haemoglobin levels were not perturbed; therefore it is unlikely that the renal dysfunction observed was severe enough to disrupt normal erythropoietin signalling and red blood cell turnover. There was no systemic disturbance of blood electrolytes, which is consistent with the renal dysfunction occurring as a secondary event to CHF, rather than as a consequence of electrolyte imbalance. The lack of changes in methaemoglobin (metHb) or fraction of carboxyhaemoglobin (fCOHb) levels indicates that the oxygenation potential of the whole blood had not been compromised. While anaemia is an important prognostic factor in cardiorenal-anaemia syndrome, it was not present in this Type-2 cardiorenal model^53,54^.

An ongoing inflammatory response in close proximity to areas of renal fibrosis has been observed up to 56 days post-LAD occlusion^39^. However, in the present study IL-1β in the renal cortex was the only cytokine other than TGF-β2, of the six measured at 90-days post-infarction, which was significantly altered. The general lack of a cytokine response may be indicative of resolution of any inflammatory response occurring prior to tissue harvest or it may be due to a tissue dilution effect as the entire medulla or cortex were assayed. IL-1β is known to play a role in infarct healing and is a modulator of pro-fibrotic AT_1_R pathways, supporting a hypothesis of resolving inflammation^55,56^.

Many clinical pharmacological interventions employed in CHF, such as beta blockers, may coincidentally reduce the development of renal injury^57^, when the myocardial damage and subsequent renal dysfunction are less severe^58,59^. Renal hypoxia, however, also triggers a cascade of intrarenal signalling which can lead to an overall change in the renal landscape. Mediators, such as catecholamines, Ang II, ROS and nitric oxide^42,60–62^ represent therapeutic targets which have shown some success in minimising disease progression when pharmacologically blocked^58,61,63–65^. CKD, however remains a major risk factor in CHF patients with reduced cardiac output and novel therapeutic targets are still needed to address the pathological activation of systemic and renal RAAS which promote electrolyte retention and plasma volume expansion^13^. Kidney injury in Type-2 CRS therefore most likely ensues from the combined effects of renal hypoperfusion, with an excessive ANS afferent output, and the systemic release of endocrine mediators and proinflammatory cytokines. This renal injury can subsequently increase posterior hypothalamic noradrenaline turnover and renal SNA elevation^66^.

This model of Type-2 CRS has enabled preliminary investigations of the interplay of these hypothalamic mediators in this chronic condition. The functional importance of an acute increase in pro-inflammatory cytokines in the PVN 14 days post-MI has been established in an animal model of HF^34^. However, pro-inflammatory cytokines were not significantly elevated in the PVN in the current study indicating this cytokine response may no longer be important by 90-days post-MI. ROS levels, however, were still elevated in the PVN and may be an important mediator of the ongoing renal injury. In a model of high salt-induced hypertension, ROS in the PVN affected renal sympathetic nerve activity, resulting in elevated plasma norepinephrine and mean arterial pressure^67^. Increased sympathetic nerve outflow in response to activation of pro-renin receptors in the PVN also appears to involve a ROS mediated signalling pathway^68^. Elevated oxidative stress in the PVN has been previously reported at 28 days post MI, probably due to AT_1_ receptor mediated NF-κB signalling resulting in an imbalance of neurotransmitters contributing to sympathoexcitation. Both the oxidative stress and extent of myocardial injury were reduced by bilateral PVN infusion of losartan^69^. Components of brain RAAS, including ACE^70^ and AT_1_R^71^, are also upregulated early following myocardial injury in several brain regions, including the PVN. Increased expression of the AngII producing enzymes renin and ACE in the PVN seen in HF studies indicates that the increased levels of AngII are produced locally and are not of systemic origin. Upregulation of AT_1_R expression in the PVN of LAD artery ligated animals may be a mechanism by which autonomic and neuroendocrine neurons are recruited in response to the stress of HF. The functional significance of upregulated brain RAAS in models of HF have been confirmed using angiotensinogen antisense RNA which attenuated cardiac remodelling, reduced sympathetic nerve activity and normalised the cardiac reflex responses^70^. The role of AngII was further confirmed by the central administration of the ACE inhibitor, enalaprilat, for 28 days following coronary artery ligation^71^. Enalaprilat attenuated renal responses to acute HF, including increased sodium retention, reduced urine sodium and volume and increased sympathetic activity with impaired baroreflex regulation. Similar results were observed for AT_1_R antagonists. These observations open up the possibility that chronic blockade of either ROS, RAAS or the resulting inducible nitric oxide synthase (iNOS) in the PVN may prevent end organ damage in hypertension or Type-2 CRS.

This model provides a robust methodology to produce  Type-2 CRS in a rat, enabling the ability to dissect, in depth, the transition from CHF, to renal injury with the potential to uncover other factor in this complex series of pathologies. However, the current study could not establish a cause-effect relationship between the PVN changes and renal dysfunction. Further studies planned to include pharmacological interventions are required. Initial studies by our group to explore the involvement of RAAS using losartan suggest a reno-protective action for this AT_1_R antagonist in this model. While analysis of cardiac contractility indices were specifically conducted in isolated isovolumic hearts at termination to remove the additional complications of cardiac innervation, future *in vivo* echocardiographic assessments conducted throughout the experimental time course would provide a valuable assessment of CHF development.

### Summary

The use of plasma cTnI to guide the positioning and extent of cardiac injury induction has enabled the establishment and refinement of a model demonstrating all the functional and morphological changes characteristic of Type-2 cardiorenal syndrome. This clinically-relevant model has also indicated a PVN involvement in the cardiorenal syndrome occurring up to 90-days post-MI, which may represent a distinct pharmacological target for this prevalent condition.

## Methods

### Animal care

Inbred male Lewis rats obtained from the University of Otago’s Animal Facility were utilised to reduce individual variation in responses. All procedures described were carried out under institutional approval in accordance with the ‘*Guidelines on the Care and Use of Laboratory Animals*’ set out by the University of Otago Animal Ethics Committee.

### Generation of post-MI chronic HF

Male Lewis rats (275–285 g) were randomly assigned to two main surgical intervention groups, non-ischaemic surgical controls (sham-ligated animals) or cardiac ischaemia by LAD coronary- ligation (LAD-ligated animals) induced, as previously described^72–74^. Animals were intubated under halothane anaesthesia and maintained on a small animal ventilator (SAR-830, Stoelting Co, Chicago USA) with a mix of 70% O_2_ 30% NO_2_ and 1–4% halothane, with flow maintained at 2.3 mL tidal volume. Core body temperatures were maintained at 37.0 ± 1 °C using a warming pad (Kent Scientific, USA). Cannulation of the femoral artery enabled blood sampling for gas analysis at the start of surgery (Radiometer ABL800 Flex blood gas analyser). Permanent coronary artery ligation was achieved using a 7–0 prolene suture around the proximal LAD artery and the occlusion verified on observation of anterior LV blanching. Bupivicaine (0.1 mL) was injected around the thoracic incision for post-operative local anaesthesia. Myocardial damage following coronary ligation was confirmed four hours post-surgery by the presence of elevated plasma levels of cardiac troponin-I (cTnI), using a commercially available high sensitivity rat cTnI ELISA (Life Diagnostics, Pennsylvania USA). Surviving LAD-ligated and sham-ligated control animals (*n* = 9 per group) were maintained for 90-days.

### Tissue and sample collection

A heparinised tail vein (IV − 0.5–1.0 mL) blood sample was taken 4 hours after the ligation, or sham ligation and the plasma separated by centrifugation (2,200 *g*, for 5 minutes, at 4 °C). A 24 hour urine sample was collected using a metabolic cage for 24 hours prior, and for blood gas analysis a blood sample was taken from the inferior vena cava immediately prior to sacrifice. On day 90, animals were weighed, heparinised (Multiparin®, 500 USP units/kg) and the hearts excised under halothane anaesthesia and rapidly perfused for isolated haemodynamic assessments^74–76^.

Both kidneys were immediately perfused *in situ* with 60 mL of tris-mannitol-sucrose (TMS) buffer containing in mM: 225 mannitol, 75 sucrose, 10 tris HCl and 0.1 EDTA (dipotassium salt) and 0.1 mM phenyl methylsulfonylfluoride protease inhibitor (pH 7.2, 4 °C). The left kidney was isolated, weighed and divided into cortex and medulla. The hypothalamus and hippocampus (control tissue) were dissected from the brain and cooled in ice-cold TMS buffer. Brain and right kidney tissues were immediately snap-frozen, ground under liquid N_2_ and stored at −80 °C for biochemical analysis. The right kidney was concurrently perfusion-fixed (4% paraformaldehyde) and prepared for histological examination. For immunohistochemistry, forebrains from an additional group (*n* = 5/group) of animals were prepared for cryosectioning according to standard protocols. Hearts from a subgroup of rats (*n* = 5/group) were arrested in diastole and *ex vivo* perfusion fixed in 0.4% paraformaldehyde. Researchers were blinded to the identity of all post-mortem samples until analysis was completed.

### Langendorff haemodynamic assessment

Hearts were cannulated through the aorta and perfused with oxygenated (95% O_2_/5% CO_2_) Krebs-Henseleit buffer at a constant pressure of 100 cm H_2_O at 37 °C, in the Langendorff mode. Using a balloon inserted into the LV, developed pressure (LVDP) was calculated as the difference between peak systolic pressure and end diastolic pressure and used to derive d*P*/dtmax, d*P*/dtmin and heart rate (HR) (Chart v5.4.2 software, AD Instruments, Castle Hill, Australia), as previously described^74–76^. LV tissue was subsequently processed for protein and enzyme analysis and stored at −80 °C. Heart weight was recorded for all animals and in a subset of rats (*n* = 4) the whole heart was dissected into individual left and right ventricles, free walls, septum and scar tissue (if present) then weighed.

### Cardiac morphology at 90-days

The ventricles were sliced coronally into four sequential blocks at 1.5 mm intervals. Blocks were fixed in Bouin’s solution (1 hr/55 °C), embedded and 10 µm sections cut and stained with picrosirius-red with Fast Green contrast, to produce a strong distinction between viable myocardium (blue green) and collagenous scar tissue (red).

### Assessment of renal function

Na^+^ concentrations were measured by flame photometry (FP20 SEAC, Italy) in duplicate urine samples prepared in a 7.5 mM Li_2_CO_3_ (Li^+^ concentration of 15 mEq/L). Endogenous creatinine in plasma and urine was analysed using a modified colorimetric Jaffe method and assessed using a Synchron Cx7 Beckman Analyser^44^. GFR was estimated (eGFR) using the calculated CrCl. Fractional excretion of sodium (FE_Na_^+^ was calculated as the (Urine [Na^+^] × Plasma [Cr])/(Plasma [Na^+^] × Urine [Cr]) × 100.

### Biochemical analyses

Renal cortex and medulla tissue samples were homogenised, using a glass-glass homogeniser, and hypothalamus and hippocampus using a sonicator (Sonics Vibra Cell, John Morris Scientific Ltd, Christchurch, NZ) in ice-cold TMS. Samples were centrifuged (4,680 *g*, 5 mins at 4 °C) and the supernatant processed for ELISA, Milliplex^TM^ and enzyme activity assays. Results were standardised to sample protein concentrations quantified using a BioRad DC Protein Assay kit (Bio-Rad Laboratories Inc., NZ)^77^. The same protein assay was used to determine urinary protein levels.

Multiplex assays (Milliplex^TM^ Rat Cytokine Assay kit, Millipore^TM^, MA, USA) for cardiac (IL-1β, IL-4, IL-6, IL-10 and TNF-α); renal (TGF-β and TNF-α); and brain (IL-1β, TNF-α and IL-6) cytokines were performed on homogenate supernatants isolated from peri-infarct cardiac tissue, renal cortex and medulla, and the hypothalamic and hippocampal brain regions, respectively. Samples were subjected to ultra-filtration (13,000 *g* for 1 hr) using 0.22 µm cellulose acetate filters (Spin-X tubes; Bonnet Equipment, Auckland, NZ) prior to assaying. The assay was conducted according to manufacturer’s instructions, with fluorescence emissions from duplicate samples and standards read on a Luminex 100^TM^ analyser (Luminex Corp., TX, USA).

Caspase-3 and −7 activities were assessed using a homogenous 7-amino-4-trifluoromethylcoumarin (AFC) caspase-3 assay kit (Anaspec EnsoLite^TM^, San Jose, CA, USA) according to the manufacturer’s instructions, as previously described^75^. The resultant generation of the AFC fluorophore after an 18 hr incubation was detected using a Gemini-EM fluorometric plate reader (Molecular Devices Corporation, CA, USA) at excitation/emission wavelengths of 380 nm/500 nm, with results expressed as relative fluorescent units per mg of protein/18 hr.

### Histology and immunohistochemistry

Collagen fibre accumulation was visualized in renal tissue using Martius Scarlet Blue stain. All qualitative histology evaluations were performed by separate researchers in a blinded manner on 5 individual sections from each block and photographed.

Coronal cryosections (16 μm) were cut (Leica, Houston, TX, USA) through the PVN region. Activated microglia were detected using anti-ionized calcium binding adaptor molecule 1 (Iba1) antibody (1:100; Abcam, Sapphire Biosciences, Hamilton, NZ) and AlexaFluor 488 donkey anti-goat IgG secondary antibody (Molecular Probes, Invitrogen CA, USA) combined with DAPI nuclear stain. AT_1_R immunofluorescence staining was carried out as above using rabbit anti-AT_1_R (1:200; Molecular Probes, Invitrogen, CA, USA) and AlexaFluor594 donkey anti-rabbit IgG (1:500; Molecular Probes, Invitrogen, CA, USA). The specificity of the primary antibodies was confirmed by the use of negative control sections in which the primary Iba1 and AT_1_R antibodies were not applied. Superoxide production, an indicator of ROS, within the PVN was determined using dihydroethidium (DHE) microfluoroscopy, as described^32^. For each treatment group, a negative control slide was prepared in parallel with no DHE applied. Following 30 mins incubation with DHE (2 µM; Molecular Probes, CA, USA) and washing, ProLong Gold Antifade with DAPI nuclear stain (Molecular Probes, Invitrogen, CA, USA) was applied to each section.

For each tissue slice, eight fields from the area of interest were randomly selected around the ventricle by a blinded observer. Iba1, AT_1_R and their respective DAPI images were photographed using a Zeiss LSM 710 confocal microscope (Carl Zeiss Vision, Germany) and overlaid using Adobe Photoshop CS3 software (Adobe). Iba1 positive cells were identified and counted using Image J (Version 1.42q, NIH), with both fluorescence and activated microglia morphology taken in to consideration with merged AT1R and Iba1 staining. Ethidium fluorescence, indicative of superoxide formation, was imaged using laser excitation at 568 nm and fluorescence product detected using a 590 nm long-pass emission filter^32^. Image analysis was performed using ImageJ and expressed against background controls (no DHE) for each sample.

### Statistical analysis

Power analysis utilising error values from previous LAD occlusion studies by the group indicated a requirement of a minimum of *n* = 8/group for functional assays and *n* = 5/group for biochemical assays. Statistical analysis was performed using Prism^TM^ 5 using a one-tailed unpaired t-test with Welch’s correction, with statistical significance taken at *P* < 0.05. Results were presented as mean ± the standard error of the mean (mean ± SEM). Statistical outliers in biochemical data were removed following a Grubbs’ test.

## Supplementary information

Supplementary Information.

## Data Availability

Data and associated protocols are available on request.
