# Supplementary material for: A Clinically Relevant Functional Model of Type-2 Cardio-Renal Syndrome with Paraventricular Changes consequent to Chronic Ischaemic Heart Failure
Source: Sci Rep. 2020 Jan 27;10:1261. doi: 10.1038/s41598-020-58071-x (PMC6985167; doi:10.1038/s41598-020-58071-x)
Supplement: Supplementary file 1 — Supplementary Information. [file 41598_2020_58071_MOESM1_ESM.pdf]

A Clinically Relevant Functional Model of Type-2 Cardio-Renal Syndrome with Paraventricular Changes consequent to Chronic Ischaemic Heart Failure.

Joanne Clare Harrison[1,*], Scott Duncan George Smart[1], Emma Maria Hinemoa Besley[1], Jessica Renee Kelly[1], Morgayn Iona Read[1], Yimin Yao[1] and Ivan Andrew Sammut [1].

[1] Department of Pharmacology and Toxicology, School of BioMedical Sciences, University of Otago Medical School, Dunedin, New Zealand.

Supplementary Data

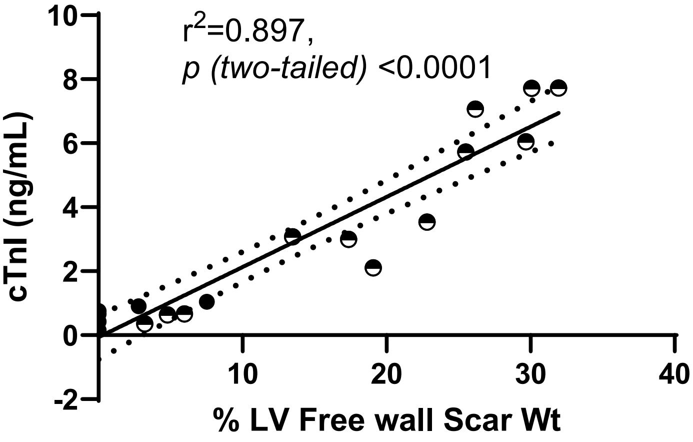

Supplementary Figure S1. Correlation between plasma cardiac troponin I (cTnI) at 4 hr post coronary ligation and left ventricular infarct size (% free wall) measured at 90 days in sham-ligated control animals (●), and LAD coronary artery ligated animals (◒). *Correlations were performed as a linear regression with Pearson's correlation (Prism v. 8.3, GraphPad software Inc.). The linear regression (———) and 95% confidence intervals (••••••••) are represented. The r^2 value and significance (p value) from the regression and correlation analyses are shown on the graph. Methods as described in main text.*

Supplementary Table SI. Haematological parameters measured by blood gas analysis. Arterial samples were obtained at the time of LADCAO, and venous samples were obtained, 90 days later, at the time of sacrifice. All values represent mean ± SEM, with no significant results obtained between sham, and LAD, animals at either time point, for any measurements.

Arterial blood gas	***Sham***	***LAD***	***p***	***Venous blood gas***	***Sham***	***LAD***	***p***
Hb ($g \cdot L^{-1}$)	143.00 ± 4.95	151.60 ± 3.99	0.1073	Hb ($g \cdot L^{-1}$)	132.60 ± 8.03	133.90 ± 3.13	0.4448
Hct (%)	43.92 ± 1.48	46.46± 1.17	0.1083	Hct (%)	40.72 ± 2.43	41.14± 0.97	0.439
pH	7.33 ± 0.03	7.34 ± 0.02	0.4197	pH	7.30 ± 0.01	7.27± 0.02	0.2039
Ca^{2+} ($mmol \cdot L^{-1}$)	0.68± 0.12	0.74± 0.16	0.3847	Ca^{2+} ($mmol \cdot L^{-1}$)	1.22± 0.07	1.08± 0.09	0.1251
Na^{+} ($mmol \cdot L^{-1}$)	139.00 ± 3.08	140.30 ± 0.97	0.3555	Na^{+} ($mmol \cdot L^{-1}$)	134.20 ± 1.24	134.30 ± 2.18	0.4867
K^{+} ($mmol \cdot L^{-1}$)	4.960± 0.47	5.143 ± 0.45	0.3930	K^{+} ($mmol \cdot L^{-1}$)	3.94 ± 0.28	4.29± 0.10	0.1504
COHb (%)	-0.44± 0.13	-0.11 ± 0.42	0.241	COHb (%)	-0.40± 0.38	-0.16± 0.39	0.3313
metHb (%)	-0.60 ± 0.25	-0.39 ± 0.32	0.3053	metHb (%)	0.28 ± 0.23	0.449± 0.24	0.3183
